# Dismantling silos: cross-sectoral response to combating child sex trafficking and online child sexual exploitation

**DOI:** 10.3389/fpsyg.2025.1625975

**Published:** 2025-07-30

**Authors:** Jennifer Martin, Kiaras Gharabaghi, Meghan Donevan

**Affiliations:** ^1^Faculty of Community Services, Toronto Metropolitan University, Toronto, ON, Canada; ^2^Department of Clinical Sciences, Faculty of Medicine, Umeå University, Umeå, Sweden

**Keywords:** child sex trafficking, online child sexual exploitation, technology-facilitated sexual abuse, child sexual abuse material, trauma, cross-sectoral practices, survivors

## Abstract

Until recently, child sex trafficking and online child sexual exploitation were treated as distinct crimes against children, leading to siloed responses from victim services, legal and policy frameworks, and law enforcement. Technology has blurred this distinction, and a cross-sectoral and multi-disciplinary response is needed as a first step to protect and respond effectively to victims. This paper presents the findings from a symposium in Toronto, Ontario, Canada in June 2024. Children’s mental health and child welfare practitioners, researchers, policymakers, child advocates, law enforcement professionals, and community members with lived experience came together to explore the current state of knowledge, policy, and practice related to technologically-assisted child sex trafficking and exploitation and gaps in responses to child victims. Using thematic analysis, we identified two overarching themes. *Sector-Specific Roles & Challenges* highlights the roles, challenges, and gaps across five key stakeholder domains. *Cross-Sector Imperatives* underscores the institutional, survivor-, and child-led leadership required to move beyond silos. We conclude with recommendations across policy, practice, and research to build coordinated, survivor-centered support systems capable of meeting the challenges of emerging technologies.

## 1 Introduction

Technology has made it cheaper and easier to recruit, advertise, and pay for child sex trafficking and online child sexual exploitation. Perpetrators are now more anonymous and have access to virtually limitless numbers of children using online spaces. Offenders who digitally record, distribute, and/or view the sexual abuse of children online are often the first to adopt and exploit technological innovations. Not only can advanced cryptographic technologies hide the identity of offenders using digital currencies, but technology allows offenders to expand their reach and ability to control children with minimal risk to themselves and on a scale that is not replicable in the physical world.

Child sex trafficking (CST) involves the recruitment, transfer, harboring and/or receipt of children for the purposes of sexual exploitation that benefits the trafficker in some way. Online child sexual exploitation (OCSE) involves the criminal depiction of the sexual abuse of children and the recording (including live web-streaming), sharing, collecting, and marketing of these images. Until recently, victims of CST and victims of OCSE were thought to be distinct from each other, requiring different research and responses from law enforcement and mental health professionals. But technology has blurred lines between these crimes, with increasing numbers of children becoming victims of both, as perpetrators continue to adapt and diversify their methods (Rimer et al., 2025). While remote learning during the COVID-19 pandemic dramatically increased the integration of technology into most aspects of children’s daily lives, it has also become ubiquitous for even young children to regularly access (if not own) a cell phone thus increasing opportunities for online exploitation through luring, sexual abuse imagery, sextortion, and ultimately sex trafficking.

Technology facilitates and expedites CST at all stages of the crime being committed. Offenders can access children via a personal digital device, especially through gaming and social media platforms. While offenders may never encounter a victim in the physical world, images of the abuse can circulate online in perpetuity. Once associated with physical abduction (Lamb, 2025), advances in technology (airtags, drones, virtual reality, tracking apps that can be installed on smartphones remotely etc.) mean that CST increasingly occurs in unremarkable settings such as a parent’s basement, involving only a laptop or mobile device; a child may be abused at home and still attend school, seeming to function as an ‘ordinary’ child. Yet victims of OCSE and CST experience complex mental health challenges, which are compounded by societal denial of these crimes and stigmatization of victims.

Some people, including professionals, may blame victims, believing that children are complicit. The sophisticated relational manipulation that accompanies CST may give the appearance of voluntary participation on the part of the child. The reality is that CST, like OCSE, is child sexual abuse: no child can consent to being sexually abused. Blame and judgement are barriers to effectively responding to the mental health needs of child victims – they perpetuate harm and can drive children deeper into the cycles of sexual abuse associated with these complex crimes (Jonsson et al., 2019). Many victims find it extremely difficult to disclose abuse and may fear being judged or criminalized, so professionals must develop specific skills to engage with and support child victims (Jones, 2025).

Despite exponentially increased risks to children and youth through technology, responses to CST and OCSE remain uncoordinated and siloed. Both crimes are global issues with lasting effects on children, families, and communities, but responses typically fail to comprehensively address multi-level issues and are instead implemented in *ad hoc* ways across sectors. For example, separate initiatives in multiple jurisdictions have focused on law enforcement, prosecution of perpetrators, safety-oriented community programs, and clinical practices for victims without coordination of knowledge or approaches across jurisdictions (Jones, 2025). Another issue is that training and awareness campaigns in settings such as schools, while necessary, further perpetuate siloed approaches by privileging particular settings or contexts. Research has confirmed that teachers and other professionals in law, education, and social and health services lack requisite knowledge and skills, and are hindered by the lack of coordination of prevention, intervention, and policy efforts (Jones, 2025; Martin and Slane, 2022; Martin et al., 2022; Slane et al., 2021; Martin et al., 2020; Rimer et al., 2018; Martin, 2016).

Organizational, sectoral, and cross-sectoral strategies are urgently needed. Effective responses will require that international scholars, experts, and people with lived experience work together to clarify and address the impact of new technologies on child victims. To address this need, we held a two-day symposium at Toronto Metropolitan University in June 2024 to explore the current state of knowledge, policy, and practice related to the role of technology in CST and OCSE and identify gaps in responses to the mental health needs of child victims. This paper reports on findings from the symposium and offers recommendations for evidence-based frameworks and programs to guide victim services and strengthen cross-sector collaboration.

## 2 Materials and methods

The symposium invited researchers, policymakers, child advocates, practitioners, community members with lived experience, and others representing various sectors and communities to exchange knowledge about technologically-assisted child sexual abuse. It sought to share research, policy, and practical knowledge relating to the complex factors involved in CST and OCSE across sectors and disciplines as well as identify priorities for future research, policy, and practice. It also provided opportunities for knowledge exchange related to interventions and response strategies with victims, with an emphasis on the devastating effects of these crimes on victims.

The symposium offered an opportunity to develop new professional, social, community, and scholarly collaborations among those present and provided unique opportunities for interdisciplinary engagement between approximately 75 local, national, and international experts from law and law enforcement, journalism, technology and cyber security, clinical practice and trauma intervention, banking, policy, and institutional leadership as well as those with lived experience.

Over the two-day symposium, plenary sessions, multidisciplinary panels, and round-table discussions fostered reflection and consolidated participants’ collective knowledge and insights. The overarching topics covered over the 2 days were informed by previous research and emerging trends identified through community partnerships. Round-table discussions were guided by open-ended questions designed to elicit current knowledge, practices, and gaps in responses to CST and OCSE. Participants documented their insights on flipcharts before presenting to the entire room. Two research assistants took notes during plenaries and panels, and all round-table flipchart notes were collated and annotated.

The authors immersed themselves in the typed notes and flipcharts and conducted thematic analysis (Braun and Clarke, 2006). The first author performed the initial coding and grouped data into candidate themes. All authors subsequently reviewed, refined, and approved the final themes and the manuscript.

## 3 Findings

During thematic analysis, we identified two overarching themes. The first, *Sector-Specific Roles & Challenges*, explores roles, limitations, and gaps across five key stakeholder domains—law enforcement, banking and finance, hospitality and tourism, journalism and media, and victim services. The second, *Cross-Sector Imperatives*, highlights the critical levers needed to move beyond silos: institutional leadership in policy, education, and research, alongside survivor- and child-led practice.

### 3.1 Sector-specific roles and challenges

#### 3.1.1 Law enforcement

Attendees explored how law enforcement and prosecution related directly to criminology and the intersections of CST and OCSE within youth criminal justice and community and social service settings like foster care, group homes, and custody facilities. They learned how CST and OCSE interconnect with technology, specifically cyber-safety, cybersecurity, and finance. Another focus of discussion was legal frameworks within Canada and globally related to CST and sexual exploitation generally, and OCSE specifically. Regarding the latter, the law is still in developmental and exploratory stages and requires innovative collaboration across sectors. CST is one of the most underreported crimes globally and is the second largest illicit industry in the world, second only to the illegal drug trade (Azzopardi et al., 2023). It is increasingly being directly tied to the internet as abusers exploit children with little risk to themselves (Ibrahim, 2022). OCSE involves the criminal depiction of sexual abuse of children and the recording, sharing, collecting, and marketing of these images. Millions of images of child sexual abuse involving hundreds of thousands of children are circulating online: law enforcement agencies have seized 2.5 million images in single collections alone, and from 2014 to 2020 Cybertip.ca, Canada’s tipline for reporting the online sexual abuse and exploitation of children, processed more than 4.3 million reports.

The internet has increased the customer base and the pool of potential victims, and livestreaming the sexual abuse of children has added to the problem. By livestreaming, perpetrators can abuse and exploit children from anywhere with little to no risk because it bypasses digital markers that law enforcement officials use to catch offenders who are downloading, sharing, or saving child sexual abuse imagery on computers or in the cloud. All that is required is internet access and a webcam or video recorder, making this form of online exploitation very difficult to trace. Perpetrators may also use pre-paid, disposable, or pay-as-you-go mobile phones to text information; send images of victims; communicate directly with other offenders in real-time without the restriction of physical location; avoid tracing through hotel/motel records; and they may use home-sharing technologies to move victims around quickly. Disposable mobile phones can also be programmed to transmit false identification, providing a high level of anonymity, and making it more difficult for law enforcement to obtain incriminating evidence for prosecution.

#### 3.1.2 Banking and finance

Throughout the symposium, attendees emphasized the critical need to engage in partnerships with financial institutions to help identify and prosecute perpetrators. While not traditionally linked to child protection, finance institutions play a pivotal role in enabling, and in turn also disrupting, CST and OCSE. Embedded in the current state of knowledge on CST and OCSE from the perspective of bankers is the observation that money is the primary motivation for perpetrators. This is an often-overlooked perspective among the professional communities responding to CST and OCSE. Perpetrators are more typically understood as sexually deviant criminals, motivated by the sexual nature of the crime. Instead, the commodification of children can transcend any moral hesitancy to monetize child sexual abuse.

Perpetrators can exchange money through online currency platforms that are difficult to trace (O’Brien and Li, 2020), often using multiple institutions to hide funds (England, 2023). They may create virtual identities to send or accept money to specific accounts using digital currency, online payment systems, credit cards, or wire transfers to make payments, and launder profits through crypto currencies. Because financial gain is the central motive of much CST and OCSE, financial institutions are almost always involved at some level (England, 2023). Thus, symposium attendees reinforced the importance of not limiting the response to CST and OCSE to distinguishing between right and wrong noting that CST and OCSE are fully integrated aspects of economic exchange and operate according to market principles – similar to the illicit drug trade or international weapons trade. Symposium attendees agreed that financial institutions must collaborate across sectors to identify, detect, and disrupt the sexual exploitation of children and that only by working together will we be able to curb this crisis.

#### 3.1.3 Hospitality industry

Audience members learned about emerging research that has centered the role of the hospitality industry. The tourism, travel, and hospitality industries are very closely linked to trafficking: hotels, motels, Airbnb and vacation rentals can be used for CST. Big cities, vacation areas, and sporting events increase demand for commercial sexual acts, leading to the exploitation and abuse of children by regional and foreign tourists (Kyriazi, 2022; Polaris, 2018). The tourism industry’s privacy and confidentiality standards are valuable to perpetrators (Kyriazi, 2022). In July 2018, an American-based anti-trafficking NGO reported that 75% of trafficking survivors had encountered a hotel and/or motel while being trafficked (Polaris, 2018). It is vital to collaborate with experts in the hospitality and tourism industries to share crucial knowledge and strengthen commitments to prevent CST and OCSE (Azzopardi et al., 2023).

#### 3.1.4 Journalism and media

The symposium addressed the critical role that the media plays in educating people – correctly or incorrectly – about the many forms of CST and OCSE (Astra, 2023). Audience members discussed how journalists play an important role in creating the narrative of OCSE and CST, which in turn informs real and perceived issues of public safety. Journalists need to weigh what is necessary for informing community education and action, and what might be harmful or re-traumatizing for victims; at present, some experts hesitate to collaborate with journalists given their lack of nuanced understanding and targeted training. At the same time, increased public awareness can improve the detection of early warning signs, so investigative journalism can be leveraged as an effective response.

Organizations including the UN have called for the need to ensure that journalists and management at media outlets are adequately informed about these issues (Astra, 2023). They must be aware of ethical considerations when investigating and reporting: words have power, and it is imperative to use accurate terminology and language as victims are at risk of re-traumatization at virtually every stage of encounter (Greijer and Doek, 2025; Martin, 2014, 2015; Martin and Alaggia, 2013). Journalists also make choices about who they represent as victims and who remains invisible, the different ways that some victims are presented strictly as victims while others are partially blamed for their victimization (such as Indigenous girls and young women), and the degree to which the narrative presented either encourages constructive vs. panicked responses and a retreat to ‘taking care of one’s own.’ Indeed, the narrative presented can move the issues of CST and OCSE into the realm of criminality on the one hand, giving rise to a societal demand for punishment for the perpetrators, or into the realm of public health, promoting much broader societal responses in which responsibilities and accountabilities are shared across sectors.

#### 3.1.5 Victim services

Participants discussed the urgent need to improve victim identification and care. Primary healthcare was identified as a space of enormous intersection between victims of CST and OCSE, and yet the system is currently failing victims. While Canadian data is limited, a US study found that 87% per cent of sex trafficking victims were known to have visited a healthcare provider at least once during the time they were held captive, yet this opportunity to identify victims was often missed (Lederer and Wetzel, 2015).

Scholars are reaching a consensus that the disciplinary contexts of traditional professional fields related to clinical interventions (social work, psychology, and mental health nursing) are currently not equipped to design and implement meaningful and evidence-informed care for victims. Symposium participants emphasized that such interventions must be highly tailored to a child’s circumstances, including their stage on the exploitation continuum. According to Richardson (2023), sexual exploitation is characterized by four stages:

1. At Risk: Children face heightened vulnerability to exploitation. Factors that increase their risk is being in care or homelessness, experiencing family disruption or abuse, alcohol and drug use, racialization, and/or are developmental disabilities. Vulnerability increases further if a family member or acquaintance is involved in the sex trade. Among the most vulnerable are Indigenous children and youth who experience the legacy of colonization and resulting barriers.
2. Transitioning In: Children become socialized into the culture of the sex trade, often via a specific trafficker or gang. Initially, the child may alternate between being sexually exploited and a more conventional way of life, e.g., alternating between group homes/shelters and the street, exchanging sex for shelter, food, drugs, and alcohol. They may start and/or increase reliance on substances, and over time they are more absent from school or other organized programs. During this stage children may start to use and understand the culture of the sex trade (language, behaviors, norms, values) and start aligning their values and beliefs while adopting more adult-like ways of engaging in social interactions. As their familiarity with the culture increase, they may start to defend the sex trade as a viable option. Finding acceptance with those who share the culture leads them to spend increasing amounts of time with them, while decreasing their connections with family and mainstream friends.
3. Entrenched: Children who are entrenched in the sex trade associate primarily with others in the culture; they have likely rejected their family and developed a new “family” within the sex trade. They completely understand and use all cultural components of the sex trade, its rules, and key players. Their lifestyle becomes defined by the culture and despite awareness of the negative components of the trade (violence to them or by them; daily substance use; daily sexual exploitation), will defend the culture to others and typically do not view themselves as exploited. Many have money, drugs, and/or clothing and other possessions they would not otherwise have.
4. Transition Away: Over time, children may become more aware of the negatives associated with the sex trade and start to talk about and internalize them. They may decrease their alcohol and drug use, and become less accepting of the violence within the trade. They may start to work with outside resources for help to exit, start to reconnect with their family and culture, and start to emotionally move away from the sex trade.

Given these distinct yet interconnected stages, the complexity of harms, and the intersections of violence that shape the experiences of CST and OCSE, effective responses cannot and must not be developed according to a “one-size-fits-all” model. At present, professionals do not differentiate between children at these very different stages – children at each of these levels of harm will respond very differently to intervention. Programs that address those who are transitioning in will fail those children who are entrenched or trying to get out. Children who are taken into temporary care or custody may be placed at severe risk and left only with the option of returning to offenders for their own safety (real or perceived) upon release. This can contribute to children being blamed for their abuse and the illusion that the child is “choosing” the sex trade.

A tailored response must also recognize the intersectional vulnerabilities that not only heighten a child’s risk of exploitation but also obstruct their access to help and protection. For example, Black and Indigenous girls are disproportionately targeted by perpetrators for exploitation (Saar et al., 2015). Black girls are also more likely to be arrested for “prostitution” – instead of being treated as victims: they may be criminalized, blamed, and even subjected to harsh interventions by the justice system including incarceration (Azzopardi et al., 2023; Cook et al., 2022). Indigenous children are also particularly at risk due to structural factors including the legacy of colonialism: trauma, abuse, and disconnection from family and community, and the need for social connection, belonging and security – all of which are exploited in the grooming and recruitment process (Hodgins et al., 2023). In addition, experiences of precarious housing and employment and transitioning out of the child welfare system place Indigenous youth in especially difficult situations due to the lack of available supports and programming, especially ones that are culturally appropriate.

These vulnerabilities are also experienced by queer, non-binary, trans, and Two-Spirit people, who often experience isolation from family, community, and mainstream society. While there is a significant lack of data on the specific needs and barriers affecting LGBTQ+ and Two-Spirit Indigenous youth, existing research indicates that queer and non-binary young people are disproportionately affected by sexual violence, abuse and sex trafficking (citing an 85% prevalence of sexual violence; Sovereign Bodies Institute, 2021). Finally, discrimination, stigma, stereotyping, and prejudices against people with disabilities put them at increased risk of trafficking and exploitation, as they feed into and reinforce the idea that they are less worthy of human rights protections and easier to abuse with impunity.

Current interventions seldom address these layered vulnerabilities and typically prioritize Eurocentric knowledge and ways of being, which can cause additional harms for Indigenous and other equity-seeking groups and fail to deliver culturally safe, trauma-informed support that meets their specific needs.

Beyond direct services for victims, most treatment programs do not include parallel treatment and support programs for parents and families who are typically also blamed. Non-offending parents and families need intense support for their own trauma so that when possible, the child can return home. Most existing programs have low expectations about exploited children returning home and there are currently no consistent evidence-based programs provided for caregivers of sex trafficked children (Martin, forthcoming). Current efforts are limited to *ad hoc* campaigns and apps aimed to keep children safe online, which are ineffective once an offender targets a child.

Attendees also noted how a lack of understanding among service providers about the differences between ‘traditional’ child sexual abuse and technologically-assisted abuse further undermines victim response as well as law enforcement and treatment/clinical interventions aimed at perpetrators. It is critical to understand how to respond to children who are victims of both CST and OCSE, specifically the unique complexity of the harms, and how to tailor best practices.

### 3.2 Cross-sector imperatives

#### 3.2.1 Institutional leadership: coordinated policy, education, and research

Participants agreed on the urgent need for professionals to know how to respond to victims of CST and OCSE and to develop the interprofessional capacity required. Research indicates that targeted education and specialized training can significantly increase effectiveness in identifying and supporting child victims (Interiano-Shiverdecker et al., 2022; Martin et al., 2022; Martin et al., 2020; Martin, 2016; Martin and Alaggia, 2013). Medical professionals, law enforcement, child protection, education professionals, and other service providers require comprehensive, trauma-informed training to help them recognize the various physical, behavioral, emotional, and psychological indicators associated with sexually exploited children (Jones, 2025; Martin, 2015, 2016). However, no programs are currently offered at Canadian universities to educate students about OCSE and CST, while *ad hoc* training remains limited and inconsistent. Jones (2025) emphasizes that without meaningful structural and systemic change, available services in Canada will continue to fail victims. These limitations point to the need for systemized and specialized training that is trauma and violence-informed, community-based, and directly informed by survivor voices and addresses the trans-sectoral nature of CST and OCSE.

Symposium attendees also expressed concern that no comprehensive framework is currently available to guide professionals working with child victims in day-to-day practice; to inform laws, procedures, and practices to ensure they fully respect the rights of child victims; or to help governments, NGOs, and community-based organizations design and implement effective legislation, policy, programs, and practices. Little empirical research has focused specifically on these crimes and most summaries of how technologies are involved rely on legal reports and media stories (Martin et al., 2020). The situation has not changed much in the years since Jaffer and Brazeau (2011) stressed the need for better, more reliable, and more easily available research and statistical information and specifically called for stakeholders to share knowledge, research, and best practices. Throughout the symposium, attendees made it known that the technologically-assisted sexual abuse of children is not just a law enforcement issue that will be resolved through the prosecution of offenders. It is a global public-health crisis that requires cross-sector professional training, a unified policy-practice framework, and ongoing research to track emerging trends and evaluate the most effective interventions.

#### 3.2.2 Survivor and child-led practice

The symposium attendees stressed the imperative of centering all responses to CST and OCSE around the voices of those with lived experience. These voices have remained largely absent and not been invited into interprofessional and transdisciplinary education, practice, and policy discussions. We must work collaboratively with those who have lived experience of being sexually exploited and/or trafficked. Many agencies employ survivors in short-term, part-time or entry-level positions but do not invest in them to build their capacity so they can move into full-time, permanent leadership roles (Richardson, 2023). The voices, perspectives, knowledge, insights, and experience of those with lived experience is integral to this work, so we cannot and should not do it without them (Richardson, 2023). Peer support is a key principle of trauma-informed care for survivors of CST; those with lived experience have a deeper understanding, connection and non-judgmental acceptance, so they may help survivors who have lost faith in other care providers and systems (Azzopardi et al., 2023). Service systems cannot follow the same patterns they have followed in the past in relation to the participation of those with lived experience. Common practice has been to invite one such person to organizational boards of Directors, or to provide feedback on program designs.

Children and youth must also have a seat at the table and directly shape our efforts. Not only are children entitled under Article 12 of the UNCRC to express their views and have them taken seriously in decisions that affect them; they also often hold the most up-to-date knowledge of emerging exploitation platforms, grooming tactics, and interventions that work best at each stage of the sexual exploitation continuum (Richardson, 2023). Ideally, youth across the four stages—“At Risk,” “Transitioning In,” “Entrenched,” or “Transitioning Away” —should be consulted. Even non-victimized peers can offer valuable insights into young peoples’ realities, for example how popular culture masks exploitation, how to spot early warning signs, and how to design effective prevention and support strategies. Children must be recognized as key stakeholders, fully capable of contributing to adult efforts to protect them (Collins, 2016).

Being survivor- and child-informed entails intentionally and systematically seeking and integrating meaningful input from a diverse community of human trafficking survivors and children to inform program or project development, implementation, and evaluation so that it accurately reflects the views, needs, and interests of the population served (National Human Trafficking Training and Technical Assistance Center, 2023). Given the enormous impact of CST and OCSE on young people, service systems must work to create a constant, impactful, and collective space for survivor and child voices and leadership in responding to these crimes and in supporting child victims. The task is to build survivor and youth networks that have resources and capacity to lead fully integrated and cross-sectoral responses to the issues. Tokenism, which has been a frequent approach in human service systems in particular, will not only not work, but can potentially be harmful and result in misguided approaches to the needs of individual victims. Including survivor and youth voices—for example through advisory committees, co-designed research, and co-lead policy initiatives—is not optional; it is essential.

## 4 Limitations

This study has two main limitations. First, the structure of the symposium, centering around specific themes and emerging trends, inevitably shaped the discussions and insights that emerged. Although this design fostered in-depth discussion, there may be other topics that were missed or unacknowledged. The breadth of stakeholder representation mitigates but does not eliminate this risk. A second limitation was the absence of child participants at this event. While several attendees were adult survivors of CST and OCSE, future convenings and research should actively include child and youth voices—drawing on both victim-survivors and non-victim peers—to ensure that policies, programs, and clinical responses are genuinely informed by those they are intended to protect.

## 5 Conclusion

The symposium was a rare moment of collaboration across highly differentiated sectors. It served as a collaborative first step in effectively responding to CST and OCSE. We synthesized the collective wisdom of all those present, and found that the core observation was that all steps being taken across sectors are helpful in some respects, but the deep and embedded presence of technology has massive implications for rapid evolution and change in the structure, process, and dynamic of CST and OCSE, so we cannot act in siloes.

Combating CST and OCSE requires an unprecedented level of speed, resolve, collaboration, and real-time knowledge and information. Those seeking to exploit children are advancing their mastery of technology as a tool to optimize their outcomes. Children themselves are advancing their technological capacities at the same time, inadvertently generating more accessible spaces and mechanisms for exploiters and abusers. It is a perfect storm: the financial incentives to engage in CST and OCSE are ever expanding, as is the market for children to gain new online skills and technological capacity. All of this is unfolding at a time when generative artificial intelligence is entering the mainstream, exponentially amplifying opportunities for exploitation and victimization.

New models are urgently needed to generate forms of collaboration that are targeted and based on real time information and knowledge. We must develop new research methodologies, new ways of generating and analyzing data, and increased capacity to integrate technology into professional activities such as clinical practice. CST and online exploitation are not contained by national or geographic boundaries, so trans-sectoral and trans-disciplinary collaboration is required to move beyond regional and national contexts and find ways to do this work transnationally and globally. This work will include finding new ways of connecting across diverse legal systems, responding to issues of technology and privacy that vary across jurisdictions, and developing the necessary relationships to circumvent delays resulting from bureaucratic impediments to real time collaboration and information sharing.

At the minimum, what is needed is a massive mobilization of a professional community united in its resolve to undo the extraordinary manifestation of child sexual abuse in the forms of CST and OCSE. This will require new conversations, humility that allows us to accept the expertise of survivors on an equal level as credentialed expertise, and determination to move into the uncertain and ever-evolving world of technology.

The knowledge shared at the symposium provided new impetus to create anchors for mobilizing in response to CST and OCSE. Universities can play their part, because they are generally stable, well- resourced, and technology-savvy spaces. Therefore, one outcome from the symposium will be the establishment of a graduate degree specifically focused on CST and OCSE at Toronto Metropolitan University. Another will be the establishment of a research center connected to that graduate degree and innovating in methodological approaches to generating the kinds of trans-disciplinary, interprofessional, and real-time knowledge we need. This includes the knowledge embedded in non- European knowledge systems such as Afrocentric and Indigenous epistemologies, wisdoms, ceremonies, and spiritualities, and it also includes the expert knowledge held by survivors. The time has passed for *ad hoc* and siloed responses. A new innovative paradigm is needed to combat the innovative technologies currently being leveraged by perpetrators.

## Data Availability

The raw data supporting the conclusions of this article will be made available by the authors, without undue reservation.
